# Challenges for the Development of Extracellular Vesicle-Based Nucleic Acid Medicines

**DOI:** 10.3390/cancers13236137

**Published:** 2021-12-06

**Authors:** Naoya Kuriyama, Yusuke Yoshioka, Shinsuke Kikuchi, Akihiko Okamura, Nobuyoshi Azuma, Takahiro Ochiya

**Affiliations:** 1 Division of Molecular and Cellular Medicine, Institute of Medical Science, Tokyo Medical University, 6-7-1 Nishishinjuku, Shinjuku-ku, Tokyo 160-0023, Japan; kuriyaman@asahikawa-med.ac.jp (N.K.); yyoshiok@tokyo-med.ac.jp (Y.Y.); okaki3313@naramed-u.ac.jp (A.O.); 2 Department of Vascular Surgery, Asahikawa Medical University, 1-1-1 Midorigaokahigashi2-jo, Hokkaido, Asahikawa-shi 078-8510, Japan; kikuchi@asahikawa-med.ac.jp (S.K.); nazuma@asahikawa-med.ac.jp (N.A.)

**Keywords:** nucleic acid drug, drug delivery system, extracellular vesicles, exosome, aptamer

## Abstract

**Simple Summary:**

The development of nucleic acid drugs has progressed in recent years, especially in the field of cancer therapy, where there has been considerable progress in the development of siRNA-, antisense oligonucleotide-, and miRNA-related drugs. Extracellular vesicles are expected to play a pivotal role as a drug delivery system for nucleic acid drugs. By conjugating EVs with proteins, antibodies, or chemical antibodies called aptamers that specifically bind to cancer, EVs can be effectively delivered to tumor tissues and cells. This review summarizes the latest findings, serving as a bridge to the clinical application of nucleic acid drugs in cancer therapy.

**Abstract:**

Nucleic acid drugs, such as siRNAs, antisense oligonucleotides, and miRNAs, exert their therapeutic effects by causing genetic changes in cells. However, there are various limitations in their delivery to target organs and cells, making their application to cancer treatment difficult. Extracellular vesicles (EVs) are lipid bilayer particles that are released from most cells, are stable in the blood, and have low immunogenicity. Methods using EVs to deliver nucleic acid drugs to target organs are rapidly being developed that take advantage of these properties. There are two main methods for loading nucleic acid drugs into EVs. One is to genetically engineer the parent cell and load the target gene into the EV, and the other is to isolate EVs and then load them with the nucleic acid drug. Target organ delivery methods include passive targeting using the enhanced permeation and retention effect of EVs and active targeting in which EVs are modified with antibodies, peptides, or aptamers to enhance their accumulation in tumors. In this review, we summarize the advantages of EVs as a drug delivery system for nucleic acid drugs, the methods of loading nucleic acid drugs into EVs, and the targeting of EVs to target organs.

## 1. Introduction

Extracellular vesicles (EVs) are phospholipid bilayer membranous vesicles and are generated by almost all types of mammalian cells as a cell-to-cell communication tool [1]. EVs carry various nucleic acids, proteins, and lipids inherited from the cell of origin. EVs have been found in body fluids such as blood [2], urine [3], saliva [4], ascites [5], pleural effusion [6], cerebrospinal fluids [7], and amniotic fluids [8]. According to the International Society for Extracellular Vesicles (ISEV), EVs can be categorized into three main subtypes based on their size and biology: exosomes, microvesicles (MVs), and apoptotic bodies (ABs). Exosomes are approximately 100 nm in diameter and are the smallest type of EV. Exosome-specific surface markers, such as tetraspanins (CD9, CD81, CD63, flotillins), integrins, and heat shock proteins (HSP) 70 and HSP90, have been identified by Western blotting and enzyme-linked immunosorbent analysis [1,9,10]. Exosomes are formed in multiple steps. Early endosomes are formed through invagination of the plasma membrane [11]. The fusion of early endosomes results in the formation of late endosomes and multivesicular bodies (MVBs) during the maturation process, and intraluminal vesicles (ILVs) are formed by the invagination of the endosomal membrane into the lumen [12]. Some of the formed MVBs bind to lysosomes and are degraded, but other fusions with the plasma membrane release ILVs into the extracellular space as exosomes [13,14]. MVs are a few hundred nanometers to a few micrometers in diameter and are formed by direct outward budding from the plasma membrane [1,15]. Apoptotic bodies are several micrometers in diameter and are formed when cells undergo apoptosis. Various types of EVs have been reported, but they are not yet clearly distinguished. ISEV has recommended that particles with lipid bilayers that have been released from cells be referred to as EVs, but there are no specific markers yet to distinguish between EV subtypes [1].

EVs have been found to play key roles in homeostasis and in the pathogenesis of various diseases, such as atherosclerosis [16,17], metabolic disorders [18], neurodegenerative disease [19], and malignant progression [20]. Of these, research on malignant tumors has advanced the most in recent years. Previous studies have shown that EVs are involved in the mechanisms of cancer angiogenesis, cell proliferation, immune escape, and metastasis [21]. Tumor cells educate surrounding immune system cells, fibroblasts, and noncellular components to promote tumor progression, and EVs act as a signal for these processes [22].

In addition, evidence has accumulated regarding the early detection of cancer using liquid biopsy and the treatment of malignant tumors. For example, the Exodx Prostate IntelliScore test is a noninvasive risk assessment tool for the detection of high-grade prostate cancer [23]. In addition, Hoshino et al. showed that cancer patients and healthy patients could be distinguished with a sensitivity of 95% and a specificity of 90% by analyzing plasma-derived EVs with proteome analysis and machine learning [24]. By using or controlling EVs related to cancer progression, cancer diagnosis and treatment methods have been advancing dramatically.

Nucleic acid drugs are therapeutic agents that can cause genetic changes in cells by using nucleic acids such as DNA and RNA. Nucleic acid drugs are divided into the following main categories: antisense oligonucleotides (ASOs), RNA interference (RNAi), microRNAs (miRNAs), and aptamers [25]. These categories represent innovative treatment options, but there are several problems. First, these materials need to be delivered to the target cell, and ASOs, RNAi, and miRNAs must be delivered across the cell membrane into the nucleus, which is a major therapeutic hurdle [25,26]. Second, RNAs can be phagocytosed by mononuclear macrophages and degraded by RNase in serum. Third, double-stranded siRNAs are rapidly excreted and cannot be incorporated into cells within blood vessels due to their polyanionic and hydrophilic nature [27]. Moreover, most nucleic acid drugs are taken up by the liver, which has a discontinuous sinusoidal endothelium and is a hyperperfused organ [28], and when administered intravenously, these drugs can lose their organ-targeting properties. To improve their delivery into cells, chemical modification of RNAs and their encapsulation in liposomes have been attempted, but some of the compounds have resulted in undesirable consequences, such as loss of biological activity [29] and toxicity [30].

Various methods to solve these problems have been developed in recent years; one is the use of EV-encapsulated RNA as a drug delivery system. EVs have hydrophilic membranes, low aggregation potential, and are decorated with CD47, which prevents phagocytosis by monocytes and macrophages and is known as the “don’t eat me” molecule [31,32]. EVs derived from mesenchymal stem cells or immature dendritic cells are biocompatible and less immunogenic, and patient-derived EVs can also be generated if required [33]. In addition, EVs have the innate ability to cross biological barriers such as the blood–brain barrier, which large molecules cannot cross [34]. Moreover, the bilayer membrane and nanoscale size of EVs protect their cargo from destruction by complement and macrophages, extending their circulating half-life and improving their biological activity [35]. By applying biological and chemical modifications to the lipid bilayer, artificial liposomes and EVs can carry a variety of substances and bypass the cell barrier. However, EVs have tetraspanin proteins, reflecting the origin and target cells of EVs. Further investigation of these proteins may improve tumor targeting with regard to EV-mediated drug delivery [36].

Therapeutic effects can also be added through biological and chemical modifications to the lipid bilayer. However, there are issues regarding the EV loading method and the delivery method to target organs and cells. In this review, we mainly focus on the recent findings of EV-based nucleic acid drugs, EV loading methods, and EV delivery methods to target organs in malignant tumors.

## 2. Therapeutic Ability of Oligonucleotides Encapsulated EVs

**Figure 1:**
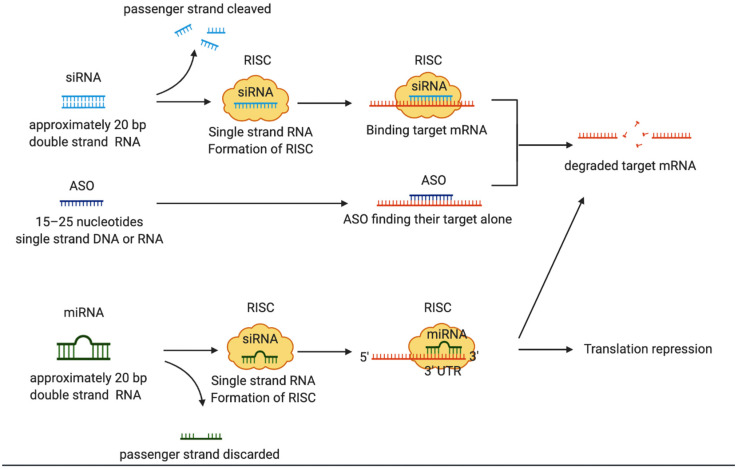
Mechanism of gene regulation by oligonucleotide therapy. SiRNA is a double strand RNA of about 20 bp, and the passenger strand is degraded followed by the formation of RISC. SiRNA draws target mRNA into RISC and degrades the mRNA. ASO is a 15–25 nucleotide single strand RNA that acts alone on mRNA and degrades it. The miRNA is a double strand RNA of about 20 bp. The passenger strand is discarded followed by the formation of RISC. miRNAs bind to the 3′UTR of mRNAs to regulate translation and degrade mRNAs.

Oligonucleotide therapy has focused on downregulating target genes via the transfection of cells and is a promising therapeutic modality for various diseases [25] (Figure 1). Some oligonucleotide therapies have been approved by the United States Food and Drug Administration (FDA) and are mainly used for treating genetic disorders [25], but they are expected to be applied in cancer treatment in the future. In Section 2, we discuss EV-related oligonucleotides used for cancer treatment. EV oligonucleotide loading methods are discussed in Section 3.

### 2.1. siRNAs

siRNAs are double-stranded RNAs of approximately 20 bp that guide the RNA-induced silencing complex (RISC) to the target sequence. siRNAs then bind to the mRNA that is complementary to their sequence. As a result, the mRNA is degraded, and specific gene expression is suppressed [37]. Several studies have investigated the use of siRNA with EV as a drug carrier for the treatment of malignant tumors. Kamerkar et al. loaded siRNA against an oncogenic mutation for KRAS (a known driver of pancreatic cancer) into EVs derived from normal fibroblast-like mesenchymal cells and assessed the therapeutic effect in vivo [38]. When the EVs were administered to various mouse models of pancreatic cancer, the cancer was suppressed, and survival rates were improved. Another study generated a lung cancer xenograft model by transplanting the A549 cell line with the KRAS^G12S^ mutation into athymic nude mice. When the mice were treated twice per week with EVs loaded with siKRAS, A549 cell proliferation was inhibited in a dose-dependent manner [39]. s100A4 is a protein known to be associated with tumor metastasis. When siRNA against s100A4 was introduced into exosomes and administered to a mouse lung metastasis model, tumor growth was significantly inhibited [40]. siRNAs loaded into EVs also have potential as therapeutic candidates for the treatment of chemotherapy resistance in malignant tumors.

### 2.2. Antisense Oligonucleotides

ASOs typically consist of 15–25 nucleotide moieties and are single-stranded DNAs or RNAs with sequence complementary to a target RNA. ASOs bind to their target RNA according to base-pairing rules to form RNA-DNA heteroduplexes, and mRNA cleavage by RNase-H causes downregulation of the target gene [41]. There are few studies on the use of EV-encapsulated ASOs for the treatment of malignant tumors. One of the best-known studies used a mouse model of Parkinson’s disease to create ASOs encapsulated in exosomes and tested the therapeutic effects [42]. In the brains of Parkinson’s disease patients, a protein called alpha-synuclein shows abnormal accumulation and is thought to play an important role in the development of the disease. The authors selected four candidate ASOs targeting *SNCA* and focused on ASO4, which was the most effective in decreasing the α-synuclein protein level in vitro. EVs loaded with ASO4 showed high cellular uptake and low cytotoxicity in vitro and significantly inhibited α-synuclein aggregation. When ASO4 was administered to α-syn A53T mice, a transgenic mouse model of Parkinson’s disease, dopaminergic neuronal degeneration was suppressed, and motor function was significantly improved. In a study on malignant tumors, Xu et al. found that EVs derived from HepG2 cells and loaded with G3139, which is an ASO of BCL-2, significantly reduced BCL-2 expression in HepG2 cells [43].

### 2.3. miRNAs

miRNAs are small RNAs of approximately 20 nucleotides and are involved in a variety of physiological processes. miRNAs repress gene expression by drawing mRNAs with target sequences into the RISC in a manner similar to siRNAs. However, in contrast to siRNAs, the recognition of target mRNA by miRNA mainly occurs through base pairing between 7 and 8 bases at the 5′ end, called the seed sequence, and through complementary sequences that are mainly in the 3′ untranslated region (3′UTR) of the target mRNA [44]. miRNAs can recognize the 5′ UTR, introns, and protein-coding regions of mRNAs [45]. Therefore, miRNAs can target a large number of mRNAs, and the same mRNA can be targeted by multiple miRNAs [25,46]. EV-associated miRNAs play a major role in tumor progression through mechanisms such as angiogenesis, immune escape, tumor growth, and premetastatic niche formation [47,48]. Therefore, the regulation of miRNA expression may help to control tumor progression. In addition, the treatment of malignant tumors using miRNA-loaded EVs is a highly studied area. Kogure et al. found that EV-associated miR-584 derived from Hep3B cells downregulated transforming growth factor beta activated kinase-1 (TAK-1) [49]. TAK-1 is an upstream member of the mitogen-activated protein kinase kinase kinase family and has an essential role in tumor progression. The authors reported that EV-associated miR-584 inhibited tumor progression in hepatocellular carcinoma. Other findings showed that EVs loaded with miR-126 mimic inhibited cell proliferation, migration, and invasion in non-small-cell lung carcinoma in vitro and blocked tumor growth in vivo [50]. Mechanistically, miR-126 binds to the integrin alpha-6 (ITGA-6) 3′UTR and suppresses ITGA6. ITGA6 reportedly interacts with RPSA to promote cell migration and invasion in pancreatic cancer [51].

Evidence supporting the delivery of nucleic acid drugs using EVs has been accumulating in recent years. Table 1 summarizes the current knowledge of EV-associated oligonucleotide therapy. Nucleic acid drugs are rapidly degraded in the circulation, so chemical modification is usually necessary. However, some sequence motifs have undesirable immune responses or lead to off-target effects [52]. To avoid them, methods for efficiently loading therapeutically effective oligonucleotides into EVs and delivering them to target organs are rapidly being developed, as described below.

## 3. Loading of Nucleic Acid Medicine into EVs

Several studies have reported the usefulness of nucleic acid therapeutics, but loading them into EVs is challenging. There are two main ways of loading RNA into EVs: pre-secretion loading and post-secretion loading. This section summarizes the EV loading methods that have been reported thus far and is divided into these two categories.

### 3.1. Pre-Secretion Loading

**Figure 2:**
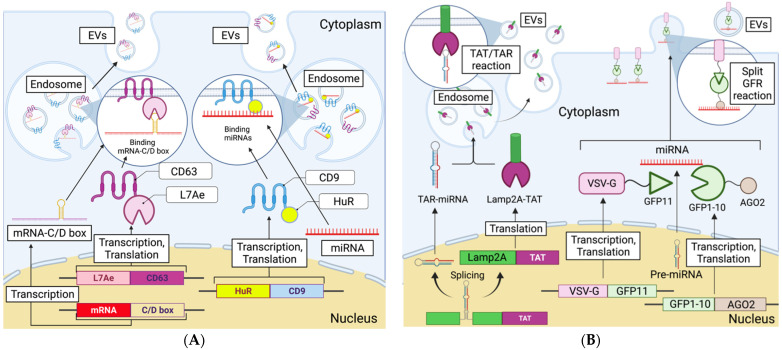
Unique and recently published pre-secretion loading methods. (**A**) Methods using tetraspanins. In the left pathway, L7Ae at the C-terminus of CD63 recognizes and binds to the C/D box of the mRNA, thereby loading the mRNA into the EV. In the right pathway, the target miRNA is overexpressed in the cell, and HuR fused with CD 9 binds to the miRNA. (**B**) Methods using other proteins. In the left pathway, the pre-miRNA loop is replaced with a TAR RNA loop and incorporated into the TAT-Lamp2A gene, loading the target miRNA into the EV. In the right pathway, VSV-G-GFP11, AGO2-GFP1-10, and the target miRNA are transfected into the parental cells. VSV-G-GFP11 binds to the lipid bilayer, and AGO2-GFP1-10 binds to VSV-G-GFP11. AGO2 introduces the overexpressed target miRNA into the EVs.

Pre-secretion loading is a method for transfecting target genes into parent cells, which increases the target gene expression in the cell, consequently leading to the target gene being loaded into EVs. Several reports have shown that small RNAs such as siRNAs and miRNAs can be transfected into parental cells using lentivirus vector or Lipofectamine and then loaded into EVs [58,61,65,67]. These methods are relatively simple ways of loading RNA into EVs, but their loading efficiencies are unknown. Recently, several new methods for the efficient introduction of target RNA into EVs via pre-secretion loading have been reported (Figure 2). Kojima et al. developed a method called EXOtic in which a target gene is transferred into the parental cell and then efficiently loaded into EVs [68]. In the EXOtic device, L7Ae, an archaeal ribosomal protein, is conjugated to the C-terminus of CD63. Next, a C/D box, which is an RNA structure recognized by L7Ae, is introduced into the 3′UTR of the target mRNA. The target mRNA can then be loaded into EVs isolated from cells transfected with these genes. This means that CD63 is an EV-specific membrane protein and that L7Ae bound to CD63 loaded the target mRNA containing the C/D box into EVs. Methods for loading miRNA have also been reported. Target miRNAs enriched in cells were shown to be efficiently loaded into EVs [69]. Target miRNA-enriched EVs were collected from cells in which the target miRNAs were overexpressed and CD9 was fused with HuR, which strongly binds RNA. In addition to this method, several new methods of transfecting RNA into cells and loading them into EVs have been reported. Sutaria et al. introduced the gene encoding pre-miR-199a into an artificial intron of the Lamp2a fusion protein, and this construct was then introduced into cells. The generated miR-199 could then be introduced into parental cell-derived EVs by using the TAT peptide/HIV-1 transactivation response (TAR) RNA interaction [70]. miR-199a was enriched 65-fold in EVs obtained using the TAT/TAR interaction compared to those without this interaction. Zhang et al. described the use of the split-GFP system and a protein called vesicular stomatitis virus G protein (VSV-G) to introduce shRNA into EVs [71]. The split-GFP system combines the protein of interest with a small GFP fragment, GFP11, and simultaneously reacts with the complementary GFP1-10 [72]. VSV-G is a fusiform viral membrane protein that is incorporated into EVs. When VSV-G-GFP11 and AGO2-GFP1-10 plasmids and PTEN-induced kinase 1 (PINK1) shRNA were transfected into 293T cells, PINK1 shRNA was loaded into EVs isolated from culture supernatants. In these methods, the target RNA is expressed in the parental cell, which produces the EVs. The target RNA is enriched in the EVs by genetically modifying the EVs to combine the protein expressed in the EVs with a molecule that has the ability to bind the RNA of interest. These methods more efficiently encapsulate the target RNA compared with simply increasing the expression of the target RNA in parental cells. They are widely used approaches to enrich molecules of interest in EVs. However, these methods cannot be used with EVs isolated from biological fluids such as patient-derived serum.

### 3.2. Post-Secretion Loading

Post-secretion loading is a method of directly processing EVs and loading them with therapeutic molecules such as RNA. This method is relatively easy to use compared to pre-secretion loading and is widely used today. Post-secretion loading methods include coincubation, electroporation, extrusion, freeze/thaw cycling, and saponin-assisted permeabilization. Among them, coincubation [73,74,75], electroporation [34,38,76], and sonication [77] have been reported as methods for loading RNA. In addition, Thakur et al. reported a method of loading EVs with shear stress using a microfluidic device called Exoload [78]. The most commonly used method at present is electroporation. However, Wahlgren et al. stated that although electroporation is useful for loading RNA, it requires optimization of the voltage, capacitance, range between electrodes in the cuvette, and concentration of siRNA and EVs [26]. Furthermore, the siRNA transfection efficiency of electroporation is approximately 25% [34]. Kooijmans et al. suggested that when siRNA is electroporated, it forms a wide range of aggregates and the substantial retention rate in EVs is less than 0.05% [79]. Owing to these issues, electroporation needs to be carefully considered, and appropriate controls are required.

As an alternative method, O’Loughlin et al. took advantage of cholesterol’s lipophilic nature [73]. They showed that siRNAs conjugated with both triethylene glycol and cholesterol can be efficiently loaded into EVs. They also optimized the method to load EVs with cholesterol-conjugated siRNA by varying the incubation time, volume, temperature, and EV/siRNA ratio. In addition to the cholesterol conjugation method, loading nucleic acids into EVs using proteins that can bind both nucleic acids and EVs has been reported [80]. EVs were incubated with the ASO for Duchenne muscular dystrophy exon-skipping therapy conjugated to CP05, which can bind CD63. As a result, the ASO was attached to the EV surface. This method does not involve loading the ASO into the EV but rather conjugates the therapeutic ASO to the EV surface.

There are several reports on RNA loading using the commercially available Exo-fect kit [39,55,81,82]. de Abreu et al. compared using the Exo-fect kit to load miRNAs into EVs with conventional loading methods such as electroporation, heat shock in the presence of calcium chloride, saponin permeabilization, and miRNA conjugation with cholesterol [82]. Exo-fect was the most efficient among these methods, with >50% transfection efficiency. Moreover, compared with native EVs, increased uptake of Exo-fect-modulated EVs by HUVECs was detected. Cellular uptake occurred mainly through endocytosis. In particular, the dominant pathway was the dynamin-dependent pathway. In addition to cellular uptake, a decrease in the interaction between EVs and lysosomes was also observed, indicating significantly more miRNA release in the cells. However, in vivo results are not yet available for this method. Therefore, even with the abovementioned advantages, sufficient therapeutic effects may not be achieved in vivo. Although it is not a primary loading method, it is a simple procedure and can be very useful.

## 4. Tumor-Targeting EVs

**Figure 3:**
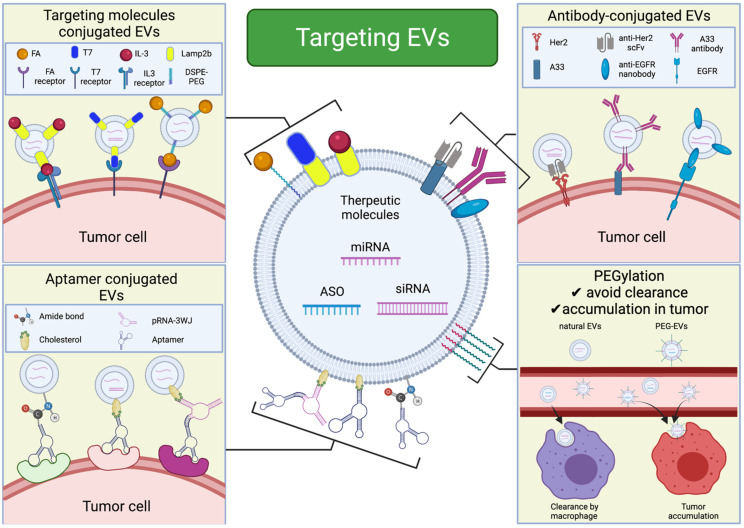
Effective targeting of EVs to tumors. Modifying EVs with various molecules, such as ligands for tumor-specific receptors, antibodies against tumor-specific membrane proteins, and aptamers, can contribute to the active targeting of EVs. EV PEGylation inhibits clearance by macrophages and increases accumulation in tumors.

Even if a target molecule is loaded into EVs and found to be effective in vitro, it is important that EVs are effectively delivered to specific organs without accumulating in healthy organs. It is also important to effectively deliver the drug to specific cells within an organ. In the following section, we focus on methods for targeting EVs (Figure 3).

### 4.1. EVs Biodistribution

As a prerequisite, it is necessary to understand how EVs are distributed after they are administered. Wiklander et al. examined differences in organ distribution according to EV dose, time after EV administration, and route of EV administration [83]. They found that when EVs were administered to mice by intravenous injection, they primarily distributed to the liver, followed by the spleen, gastrointestinal tract, and lungs. Higher EV doses resulted in decreased accumulation in the liver and increased distribution in the intestine and lung. Compared with intraperitoneal and subcutaneous administration, intravenous administration led to more accumulation in the liver and spleen. Conversely, intraperitoneal and subcutaneous administration showed more accumulation in the pancreas and gastrointestinal tract when compared with intravenous administration. In addition, Lázaro-Ibáñez investigated biodistribution of radiolabeled EVs by nuclear imaging using single-photon emission computed tomography (SPECT) and computed tomography (CT) [84]. The real-time monitoring of ^111^indium-DTPA-labeled EVs demonstrated being the most sensitive and accurate for in vivo tracking, more so than fluorescent (mCherry) and bioluminescent (Firefly and Nanoluc luciferase) proteins fused to EVs. According to these results, intravenously injected ^111^indium-DTPA-labeled EVs accumulated mostly in the liver, followed by the spleen and the kidney. Thus, naïve EVs can still accumulate in the liver, lungs, and spleen to some extent, and therapeutic effects may be obtained. However, in order to increase the accumulation of EVs in target organs or target cells, such as tumor cells, it is necessary to modify EVs in some way.

### 4.2. Passive Targeting

Passive targeting takes advantage of the physical and chemical properties of EVs as well as the anatomical and physiological characteristics of organisms. The vasculature of cancer tissue is different from that of normal blood vessels due to the hypervasculature and increased vascular permeability. Matsumura et al. reported that molecules tens to hundreds of nanometers in size, such as small EVs, that had been circulating for a long time extravasated through the fenestrated vasculature of tumors and accumulated in tumors; this is called the enhanced permeability and retention (EPR) effect [85]. Well-known anticancer drugs that utilize this effect are liposomal doxorubicin (Doxil) and nanoparticle albumin-bound paclitaxel (Abraxane) [86,87]. In other words, one way to effectively deliver therapeutics into cancer tissue is to achieve a longer circulation time in the blood vessels. When EVs are administered, they are taken up by the mononuclear phagocyte system (MPS) in the liver and spleen [88]. Positively charged nanoparticles are rapidly cleared by the MPS. In contrast, neutral and zwitterionic particles have long half-lives [89]. EVs have a slightly negative surface charge under physiological conditions [90], which suppresses their clearance by the MPS in comparison with positively charged nanoparticles [91]. In addition, small EVs express the glycosylphosphatidylinositol (GPI)-anchored regulators, CD55 and CD59, which are membrane regulators of complement [92]. This allows small EVs to escape degradation and immune responses in the blood vessels. Thus, EVs may have a longer circulation time than other artificial nanoparticles. However, one report suggested that the half-life of exosomes in circulation is approximately 2 min [93], which is comparable to that of liposomes [94]. Therefore, compared to artificial nanoparticles, EVs may have a longer circulation time in the blood, but there are few reports demonstrating this effect.

One well-known method of reducing the clearance of EVs is polyethylene glycol (PEG) conjugation, which is called PEGylation. Shi et al. used copper-64 (64Cu)-radiolabeled EVs to analyze the accumulation of PEG-modified EVs and natural EVs in vivo [95]. The PEGylated EVs significantly accumulated in the tumor and showed slower clearance by the liver compared to naive EVs. However, there are some problems with PEGylation. Once PEG is administered, anti-PEG-IgM antibodies are produced, and the PEGylated EVs are rapidly cleared by the liver after the second dose [96]. Moreover, when PEGylated EVs are administered, most are taken up by the liver and spleen. Therefore, focusing on reducing the clearance of EVs will help to improve this method of EV targeting.

### 4.3. Active Targeting

Studies have reported methods for improving tumor targeting and organ/tumor specificity by adding various modifications to EVs and taking advantage of the characteristics of the tumor microenvironment (TME). The TME is acidic due to dysregulated energy metabolism, inadequate perfusion, and uncontrolled cell growth [97,98]. One method of delivering therapeutically effective EVs to the TME has been reported that takes advantage of this acidic environment [99]. Following Mn^2+^ activation of RAW264.7 to M1 macrophages, the M1 macrophage membranes were modified with azide, and EVs were collected. These EVs were conjugated with dibenzocyclooctyne-modified antibodies against CD47 and signal regulatory protein alpha (SIRPα) through pH-sensitive linkers. In the acidic TME, cleavage of the benzoimine bond of the nanobioconjugate released anti-SIRPα and anti-CD47, which blocked SIRPα on the macrophages and CD47 on the tumor cells, thereby eliminating the “don’t eat me” signal and improving macrophage phagocytosis ability. 4T1 tumor-bearing mice treated with these EVs had reduced tumor volumes and improved survival compared to those treated with normal M1-derived EVs.

Tumor-derived EVs have organotropic tumor-homing properties. Therefore, a homing effect can be obtained by loading tumor-derived EVs with therapeutic molecules. Qiao et al. added Dil-labeled EVs derived from HT1080 cells and Dil-labeled EVs derived from HeLa cells to HT1080 cells, and the uptake of each EV was compared [100]. Compared to HeLa cell-derived EVs, HT1080 cell-derived EVs exhibited 2-fold higher uptake. Similarly, a twofold higher uptake of HeLa cell-derived EVs was observed for HeLa cells when compared with the HT1080 cell-derived EVs. The authors also investigated the cancer-targeting ability of tumor-derived EVs using nude mice bearing a subcutaneous HT1080 tumor. HT1080-derived EVs showed approximately 2-fold higher tumor accumulation than HeLa cell-derived EVs. When EVs were loaded with the antitumor agent Doxil and administered to tumor model mice, a significant decrease in tumor weight was observed. Additionally, based on the proteome array, the authors suggested that these effects indicate a link between EV surface integrin and tumor tropism. In support of these findings, Hoshino et al. showed that the subtype of integrin expressed in EVs can predict the destination metastasis [101]. Moreover, exosome-mimetic nanosystems expressing integrin α6β4 and loaded with miR-146 mimic, which have a therapeutic effect on lung cancer, were effective in vitro and in vivo, respectively [102]. The nanosystems also showed significantly reduced accumulation in the liver and kidney compared to normal EVs. This report suggested that cancer-specific integrins on EVs can efficiently reach the tumor and probably result in lower systemic toxicity. Therefore, if the expression pattern of cancer cell-specific integrin subtypes can be determined, it has the potential to enable the tumor-specific delivery of EVs.

In addition to integrins, various other molecules are being investigated for their ability to effectively deliver EVs to cancer cells. Although not malignancy-related, a well-known report showed that rabies virus glycoprotein (RVG) (which specifically binds to acetylcholine receptors) fused to the N-terminus of Lamp2b (an EV membrane protein) effectively delivers EVs to neurons, microglia, and oligodendrocytes in the brain [34]. siRNA against BACE1, a therapeutic target for Alzheimer’s disease, was loaded into this EV by electroporation and administered to wild-type mice, resulting in the effective knockdown of BACE1. A well-known cancer-related report showed the successful generation of breast cancer-targeting EVs using the GE11 peptide, which binds to epidermal growth factor receptor (EGFR) but is less mitogenic than EGF, targeting EGFR [66]. There are other reports on folate-displaying EVs that target folate receptors, which are known to be expressed on the surface of various types of cancer cells, and EVs that target HER2-expressing cancer cells [39,103,104]. As mentioned above, various methods have been reported to conjugate target peptides and antibodies to EVs for effective targeting (Table 2). However, these approaches to EV engineering are challenging. Genetically modifying EV parent cells to express the target protein on the EV surface requires viral transduction, selection, and large-scale cell cultures. Additionally, it is possible that plasmids and transgenes may be contained in EVs, and there is a possibility of horizontal gene transmission. It has also been reported that various other carcinogenic factors can be introduced into EVs when cancer cells are used as the parent cells of EVs [105]. Additionally, chemical- and affinity-based conjugation methods do not modify genes but are transient and unstable; furthermore, they are difficult to carry out because appropriate reaction conditions must be established [106].

Pham et al. developed a simple enzymatic method to bind peptides and nanobodies to EVs via covalent bonds without genetic or chemical modifications [109]. The authors used the enzymes sortase A and OaAEP1 ligase to bind proteins of interest to the surface of erythrocyte-derived EVs. These enzymes did not change the structure of EVs, and the target protein bound to approximately 80% of them. The authors conjugated EVs derived from erythrocytes and also EVs derived from THP1 cells, and they were able to conjugate EVs to proteins involved in EV targeting, such as EGFR-, HER-2-, and CD47-derived self-peptides. These methods enable the relatively easy and covalent conjugation of antibodies against tumor-specific proteins to EV surfaces without the need for genetic or chemical modifications. This may be a very useful method for creating EVs that target tumor-specific surface proteins.

### 4.4. Active Targeting Using Aptamers

Aptamers are called chemical antibodies and are single-stranded DNA or RNA oligonucleotides that fold into a three-dimensional structure to mimic antibodies. They bind specifically to their targets with high affinity and specificity. Several unique features of aptamers can be modified by chemical procedures without the need for organisms. They are suitable for large-scale synthesis, are cost-effective, have low or no immunogenicity, low batch-to-batch variation, and chemical modifications can easily be incorporated for enhanced stability and binding capacity [116]. Aptamers are generated by amplifying selected nucleic acids from a random oligonucleotide library using the polymerase chain reaction-based method known as systematic evolution of ligands by exponential enrichment (SELEX) [117]. The SELEX method can be used to develop aptamers against various cell surface molecules that are found on tumor cells and tumor-educated cells in the TME. In addition, SELEX has the ability to generate specific aptamers against target molecules without prior knowledge of the signature. The FDA has approved an anti-vascular endothelial growth factor antagonist aptamer called pegaptanib (Macugen) and a drug for age-related macular degeneration [118]. There has been accumulating evidence of EV targeting using aptamer properties in recent years. Wan et al. found that EVs conjugated with the AS1411 aptamer had an antitumor effect in vivo [112]. The AS1411 aptamer can bind to nucleolin, which is expressed on the surface of various types of cancer cells [119]. The AS1411 aptamer covalently conjugated to cholesterol-poly was grafted onto live mouse dendritic cell membranes. These cells were then mechanically extruded to generate AS1411 aptamer-conjugated EVs. Paclitaxel was loaded into these EVs by sonication, and the therapeutic effect was investigated in vivo. AS1411-EVs significantly accumulated in tumors compared to EVs without AS1411 conjugation. Moreover, AS1411-EVs loaded with paclitaxel significantly inhibited tumor growth and tumor volume. The advantage of this method is that the extrusion of approximately 10^7^ cells can create tumor-targeting EVs at an amount of 3 × 10^10^ in 1 h. Pi et al. reported a unique aptamer conjugation method utilizing the intrinsic nature of the three-way junction (3WJ) of the bacteriophage phi29 motor pRNA. pRNA-3WJ is arrow-shaped, and when cholesterol is conjugated to its tail part, RNA is displayed on the EV surface [55]. pRNA-3WJ RNA was incorporated with a PSMA aptamer, which has the ability to target prostate cancer, and the tumor specificity of PSMAapt/EVs was investigated. PSMAapt/EVs showed significant EV uptake by LNCaP cells, which are PSMA-positive prostate cancer cells. Subsequently, survivin siRNA, an inhibitor of cell apoptosis, was encapsulated within EVs and its antitumor effect was examined in a mouse model of prostate cancer. PSMAapt/EV/siSurvivin led to a significant reduction in tumor size compared with PSMAapt/EV/siScramble. There have been recent reports of EV drug delivery methods using aptamers that recognize MUC-1, a transmembrane mucin glycoprotein expressed in epithelial cells and particularly abundant in cancer cells, as well as an sgc8 aptamer that recognizes a membrane protein called protein tyrosine kinase 7 (PTK7) [114,115]. There are various known mechanisms of EV cellular uptake, such as membrane fusion, clathrin-mediated endocytosis, lipid raft-mediated endocytosis, caveolin-mediated endocytosis, phagocytosis, and micropinocytosis [12]. Zou et al. showed that aptamer-conjugated EVs were internalized via multiple endocytosis pathways, especially the clathrin-mediated pathway, which plays a major role in endocytosis; by contrast, uptake through micropinocytosis and caveolin-mediated endocytosis were not major pathways [115]. It is expected that aptamers will find clinical applications due to their relatively easy production, low immunogenicity, and high affinity and specificity. However, to date, pegaptanib is the only aptamer that has been clinically applied, and this may be due to various limitations to the actual operation of the system. To conjugate aptamers to EVs, modification of PEG linkers and cholesterol or modification using chemical reactions is often necessary [55,113,114,115]. In addition, given that aptamers may be degraded by nucleases in the serum, it may also be necessary to provide nuclease resistance to the aptamer through chemical modifications of the phosphodiester bonds between sugars and nucleotides [116]. Although there are various problems to be solved, the development of aptamers has been progressing rapidly in recent years. For example, SOMAscan is a well-known diagnostic platform that recognizes a variety of proteins [120]. By taking advantage of the abovementioned properties of aptamers, EVs conjugated with aptamers are expected to be a promising nanoplatform for the delivery of therapeutically effective molecules.

## 5. Conclusions

EVs have a lipid bilayer structure and express CD47, a membrane protein known as a “don’t eat me” signal. EVs are more biocompatible and less immunogenic than liposomes and accumulate more readily in cancer tissues than in normal tissues due to the fenestrated vascular structure of cancer tissue, known as the EPR effect. Although few studies have compared EVs with artificial nanovesicles, such as liposomes, the properties of EVs indicate that they could be useful drug delivery devices in cancer therapy. In addition, various modifications to EVs can increase their accumulation in tumors. For example, PEGylation reduces clearance by the MPS, and the addition of tumor-specific integrins or antibodies to the EV surface has been shown to increase tumor accumulation. Conjugating EVs with aptamers, also known as chemical antibodies, also increases the tumor accumulation potential of EVs. There are two methods of loading EVs. Pre-secretion loading involves the genetic modification of parental cells, while post-secretion loading involves the direct loading of nucleic acid drugs into EVs. Pre-secretion loading is a simple method, but its loading efficiency is unknown, and there are concerns about safety because it involves genetic modification. Electroporation is the most commonly used post-secretion loading method, but its loading efficiency needs to be carefully considered. Alternatively, Exo-fect is a commercially available kit that gives good loading efficiency and is a useful loading method. In general, considering the use of EVs as a drug delivery system for cancer treatment, there are various problems still to overcome, such as how to produce a large amount of EVs and how to collect them.

This review discusses the latest research on the combination of nucleic acid drugs and EVs. These EV-nucleic acid complexes might be future candidates for cancer therapy, and we hope that the technologies described herein will be used to develop novel nucleic acid drugs for anticancer therapy in the near future.

## Figures and Tables

**Table 1:** EV-related nucleic acid therapeutics.

Types of Oligonucleotide	Cargo Mediators	Cancer Type	Parent Cell	Loading Method	Function	References
siRNA	siS100A4	breast cancer	breast cancer cell	Coincubation and extrusion	Involved in various pathways	[40]
	si-c-Met	gastric cancer	HEK293T cell	Parental cells transfection using Lipofectamine	Reverse chemoresistance to Cisplatin	[53]
	siKRAS^G12S^	lung cancer	Milk	Electroporation and Exo-fect	Antiproliferative effect via silencing KRAS^G12S^	[39]
	BCR-ABL siRNA	Chronic myeloid leukemia	HEK293T cell	Parental cells transfection using Lipofectamine	Chronic myeloid leukemia cell growth in vitro and in vivo	[54]
	siSurvivin	Prostate cancer	HEK293T cell	Binding of Cholesterol to the arrowhead of pRNA-3WJ fused with siSurvivin	Inhibition cell apoptosis	[55]
ASO	G3139 (BCL-2 ASO)	Hepatocellular carcinoma	HepG2 cells	Cholesterol-conjugated ASO was loaded onto the EVs	Downregulation of anti-apoptotic Bcl-2	[43]
	Antisense miRNA oligonucleotide against miR-21	Glioblastoma	293T cells	Electroporation	Reduction of tumor size via upregulation of PDCD4 and PTEN	[56]
miRNA	miR-126	non-small cell lung carcinoma	patient serum	Exo-fect	Inhibiting tumor proliferation and migration via downregulation of ITGA6	[50]
	miR-199	Ovarian cancer	Omental fibroblast derived from ovarian cancer patients	Electroporation	Inhibition of cell proliferation and invasion via suppression of c-Met	[57]
	miR-21-sponge	Glioblastoma	HEK293T cells	Parental cells transfection using Lipofectamine	Declining cell proliferation and elevation in apoptotic rates via upregulation of PDCD4 and RECK	[58]
	miR-128-3p	Colorectal cancer	FHC cells	Parental cells transfection using Lipofectamine	Upregulation of E-cadherin and inhibition oxaliplatin-induced epithelial mesenchymal transition by downregulation of Bmi1, and decreasing oxaliplatin efflux via suppression of MRP5	[59]
	miR-335-5p	Hepatocellular carcinoma	LX2 cells	Parental cells transfection using Lipofectamine	Inhibition of hepatocellular carcinoma cells proliferation and invasion through downregulation of 13 mRNA	[60]
	miR-379	Breast cancer cells	MSCs	Lentiviral transfection of parental cells	Suppression of tumor growth via downregulate cyclooxygenase-2	[61]
	miR-26a	HepG2 cells	293T cells	Electroporation	Decreasing cell migration and proliferation via downregulation of CCNE2 and CDK6	[62]
	miR-124a	Glioblastoma	MSCs	Lentiviral transfection of parental cells	Significant reduction in viability due to abnormal lipid accumulation through silencing FOXA2	[63]
	miR-584	Glioma	MSCs	Lentiviral transfection of parental cells	Inducing tumor cell apoptosis and reducing tumor cell invasion via enhancing caspase-3 and reducing matrix metalloproteinase-2 expression	[64]
	miR-122	Hepatocellular carcinoma	adipose tissue-derived MSCs	Parental cells transfection using Lipofectamine	Increasing chemosensitivity through downregulation of CCNG1, ADAM10, and insulin-like growth factor 1 receptor	[65]
	let-7a	Breast cancer	HEK293 cells	Parental cells transfection using HiPerFect reagent	Suppressing tumor growth in vivo	[66]
	miR-146b	Glioma	MSCs	Parental cells transfection using electroporation	Reducing tumor size via suppressing EGFR and NF-κB	[49]

**Table 2:** Tumor-targeting molecules and how to load onto EVs.

Targeting Molecule	Target to	Cancer Type	How to Add Targeting Molecule	References
Antibody				
anti-Her2-scFv	Her2	Breast cancer	Binding of anti-Her2-scFv to C1C2 domain of lactadherin that can bind to phosphatidylserine	[104]
A33 antibody	A33	Colorectal cancer	EVs isolated from A33 positive LIM1215 were coated with surface-carboxyl superparamagnetic iron oxide particles with A33 antibodies	[107]
somatostatin receptor-2 antibody	somatostatin receptor-2	Neuroendocrine cancer	Coincubation of anti-SSTR Ab with 1,2-Distearoyl-sn-glycero-3-phosphoethanolamine (DSPE)-PEG-N-hydroxysuccinimide and mPEG-DSPE-EV	[108]
EGFR targeting nanobody	EGFR	Lung cancer	Simple enzymatic method to bind peptides and nanobodies to EVs via covalent bonds using Sortase	[109]
Peptide or other molecules				
c(RGDyK) peptide	α_V_β_3_ integrin	Glioblastoma	Coincubated with micelles formed by DSPE-PEG2000-c(RGDyK)	[110]
Folate	Folate receptor	Breast cancer	Coincubated with folate conjugated with DSPE-PEG2000	[103]
Folate	Folate receptor	Lung cancer	Covalently conjugation using standard stable amide chemistry	[39]
RGERPPR peptide(RGE peptide)	Neuropilin-1	Glioma	The alkyne group was conjugated with phosphatidylethanolamine on the exosome surface, and the RGE peptide with an azide group was conjugated with the alkyne group by a triazole linkages.	[111]
iRGD peptide	αv integrin	Breast cancer	Parental cells were transfected with the vector expressing iRGD-Lamp2b fusion protein	[112]
GE11	EGFR	Breast cancer	Parental cells were transfected with the plasmid containing platelet-derived growth factor receptor transmembrane domain fused with GE-11	[66]
Interleukin-3 (IL3)	IL3-R	Chronic myeloid leukemia	Parental cells were transfected with the plasmid containing Lamp2b gene fused with the IL3 gene fragment	[54]
T7	Transferrin receptor	Glioblastoma	Parental cells were transfected with the plasmid containing Lamp2b gene fused with a T7	[56]
Aptamer				
AS1411 aptamer	nucleolin	Breast cancer	Extrusion of dendritic cells labeled with Aptamer conjugated with PEGylated cholesterol	[113]
PSMA aptamer	PSMA	Prostate cancer	Conjugation of aptamer with pRNA-3WJ fused with cholesterol.	[55]
MUC1 aptamer	MUC-1	Colorectal cancer	Utilizing amine groups on the surface of EVs to bind via amide bonds	[114]
scgc8 aptamer	Protein tyrosine kinase 7	T-cell leukemia	Diacyllipid conjugated aptamer decorated onto EVs through hydrophobic interaction between the diacyllipid tail and the phospholipid bilayer of EVs.	[115]
